# Design and chemical composition of a reference phantom for ^13^C metabolic MRSI

**DOI:** 10.1007/s10334-026-01330-1

**Published:** 2026-02-12

**Authors:** Julian Schüle, Christoph A. Müller, Sebastian Lucas, Leonard Schraff, Stefan Menzel, Tobias Speidel, Ilai Schwartz, Volker Rasche

**Affiliations:** 1 Department of Internal Medicine II, Ulm University Medical Center, 89081 Ulm, Germany; 2 Core Facility Small Animal MRI, Ulm University, 89081 Ulm, Germany; 3 NVision Imaging Technologies GmbH, 89081 Ulm, Germany

**Keywords:** Magnetic resonance spectroscopy, Magnetic resonance imaging, Phantoms, Imaging, Carbon isotopes, Reproducibility of results

## Abstract

**Object:**

Development of a reproducible and long-term stable reference phantom for ^13^C metabolic MRSI, addressing the need for accessible calibration and quality assurance tools across sites and platforms.

**Materials and methods:**

The phantom was built from readily available labware (50 mL centrifuge tubes, NMR glass vials) and 3D-printed holders, designed to ensure sufficient coil loading and excellent B_0_ homogeneity. ^13^C-labeled reference solutions were stabilized by argon degassing, sodium azide addition, and airtight flame sealing, with optional gadolinium doping to tune T_1_/T_2_ relaxation. Performance was tested at 1.4 T, 3 T, and 11.7 T using ^1^H MRI, ^13^C spectroscopy, and ^13^C MRSI.

**Results:**

The reference solutions remained chemically and spectrally stable for up to 20 months under cooled, light-protected storage. Ethyl acetate provided a stable alternative to pyruvate, while gadolinium enabled the control of relaxation properties. Spectral assessment confirmed narrow linewidths, strong SNR, and reproducible peak separation across field strengths, validating the phantom’s suitability for calibration and method development.

**Discussion:**

This low-cost, open-source design provides a durable and reproducible ^13^C phantom that complements existing dynamic and metabolite-specific models. It enables long-term standardization, supports multi-center studies, and facilitates robust quality assurance in hyperpolarized and thermal ^13^C MRSI.

## Introduction

Magnetic resonance imaging and spectroscopy with hyperpolarized ^13^C-labeled compounds has emerged as a powerful tool for real-time metabolic imaging, enabling non-invasive investigations of biochemical processes in vivo. However, the quantitative accuracy and reproducibility of hyperpolarized ^13^C MRSI remains challenging due to factors such as signal decay, polarization variability, and scanner-dependent properties. To address these challenges, ^13^C-labeled reference solutions contained in MR imaging phantoms play a crucial role in system calibration and quality assurance. These phantoms contain stable chemicals and provide reproducible signals as a reference, allowing for spectral quantification, standardization across imaging platforms, and enhanced reliability of metabolic assessments.

Recent studies have highlighted the importance of such phantoms in various aspects of hyperpolarized ^13^C MRSI. A key challenge remains in ensuring reliable quantitative metrics across institutions, which has led to the development of dynamic phantoms [1–3], improved imaging sequences [7–12], and rigorous quality assurance protocols [13, 14].

A synthetic enzyme phantom enabling reproducible conversion of hyperpolarized [1-^13^C]-pyruvate to [1-^13^C]-lactate provides a controlled model of normal or pathological metabolism [1]. Similarly, a dynamic phantom further supports reproducible quantification of hyperpolarized ^13^C MR metrics and cross-site sequence standardization [2]. This was further advanced by a multi-compartment dynamic single-enzyme phantom simulating realistic biological conditions for systematic testing of ^13^C-labeled agents [3]. Wiesinger et al. employed a phantom as a stable reference alongside in vivo experiments, enabling calibration and validation of dynamic metabolic measurements under controlled conditions [4].

A crucial technical consideration is the accurate calibration of the flip angle to optimize the signal-to-noise ratio (SNR). Early work emphasized the importance of precision in flip-angle calibration for accurate metabolite quantification [5]. Building on this, a protocol to characterize the spatial profile of ^13^C surface coils was proposed to address coil sensitivity variations and support parallel imaging strategies [6].

Standardized phantoms also play a key role in pulse sequence development and validation for hyperpolarized MRSI. Several advanced acquisition strategies have improved the spatiotemporal resolution of hyperpolarized ^13^C MRSI, including balanced steady-state free precession (bSSFP) sequences with spectral suppression, SPICE dynamic imaging, compressed sensing with multiband excitation, single-shot 3D sequences, and metabolite-specific echo planar imaging [7–12]. These methods enable rapid and high-fidelity mapping of metabolites such as pyruvate and lactate, supporting both preclinical studies and sequence optimization for clinical translation.

The integrity of hyperpolarized ^13^C data also depends on robust quality assurance frameworks. Earlier studies have laid the groundwork for metabolic data validation in MR spectroscopic imaging [13]. Further, phantoms have been used for verification of simultaneous ^13^C MRSI and PET measurements without compromising PET quantification capabilities, thus indicating the system’s suitability for in vivo ^13^C MRSI and the possibility of integrated PET/MRSI metabolic imaging for clinical translation [14].

The importance of thermally polarized standardized phantoms instead of hyperpolarized solutions was pointed out by the consensus recommendations for multi-center human studies using hyperpolarized [1-^13^C] pyruvate MRI [15]. Although the use of thermally polarized phantoms was endorsed with full consensus, the field still lacks a universally accessible ^13^C reference phantom that can be easily built in any laboratory and used for routine calibration and method development.

Here, we present a robust phantom using concentrated thermally polarized ^13^C solutions. It is built from widely available materials, provides stable long-term signals without hyperpolarization decay, and can be assembled for less than €1,500. We describe its design, characterization, and potential applications, emphasizing chemical composition, relaxation properties, and reproducibility to establish a practical reference standard for preclinical and translational studies.

## Materials

The chemical compounds that were used for the production of the ^13^C-labeled reference solutions are listed in Table 1.

**Table 1:** List of chemical compounds with their respective quantities, purities, CAS numbers (supplier SIGMA-ALDRICH Chemie GmbH, Taufkirchen, Germany, or Guerbet Group, Villepinte, France)

Chemical name	Quantity	Purity	CAS number	Supplier
Sodium L-lactate-1-^13^C solution (lactate)	700 μL	99 atom % (45–55% (w/w)), 98% CP	81273–81-6	SIGMA-ALDRICH
L-Alanine-1-^13^C (alanine)	316 mg	99 atom %	21764–56-7	SIGMA-ALDRICH
Urea-^13^C (urea)	740 mg	99 atom %, 99% CP	58069–82-2	SIGMA-ALDRICH
Ethyl acetate-1-^13^C (ethyl acetate)	1000 mg	99 atom %, 99% CP	3424–59-7	SIGMA-ALDRICH
Sodium bicarbonate-^13^C (bicarbonate)	212 mg	98 atom %, 99% CP	87081–58-1	SIGMA-ALDRICH
Deionized H_2_O	2 mL (per dissolved chemical)		7732–18-5	
Gadoteric acid (Dotarem^®^)	1 μL (for 1 mL solution)		72573‑82‑1	Guerbet group

Further materials included: 5 mm outer diameter NMR tubes (Hilgenberg GmbH, Malsfeld, Germany), and 50 mL plastic centrifuge tubes (Falcon, VWR International GmbH, Darmstadt, Germany). The following devices were used during the production and assembling of the phantoms: a vacuum pump (PC 3002 Vario, VacuuBrand GmbH + Co. KG, Wertheim, Germany); Bunsen burner (VersaFlame, DREMEL, Breda, Netherlands); 3D-printer (Bambu Lab X1-Carbon, Makerworld Singapore PTE. LTD., Singapore), and a Precision balance (LP-4202i, VWR International BVBA, Leuven, Belgium)

## Methods

**Fig. 1:**
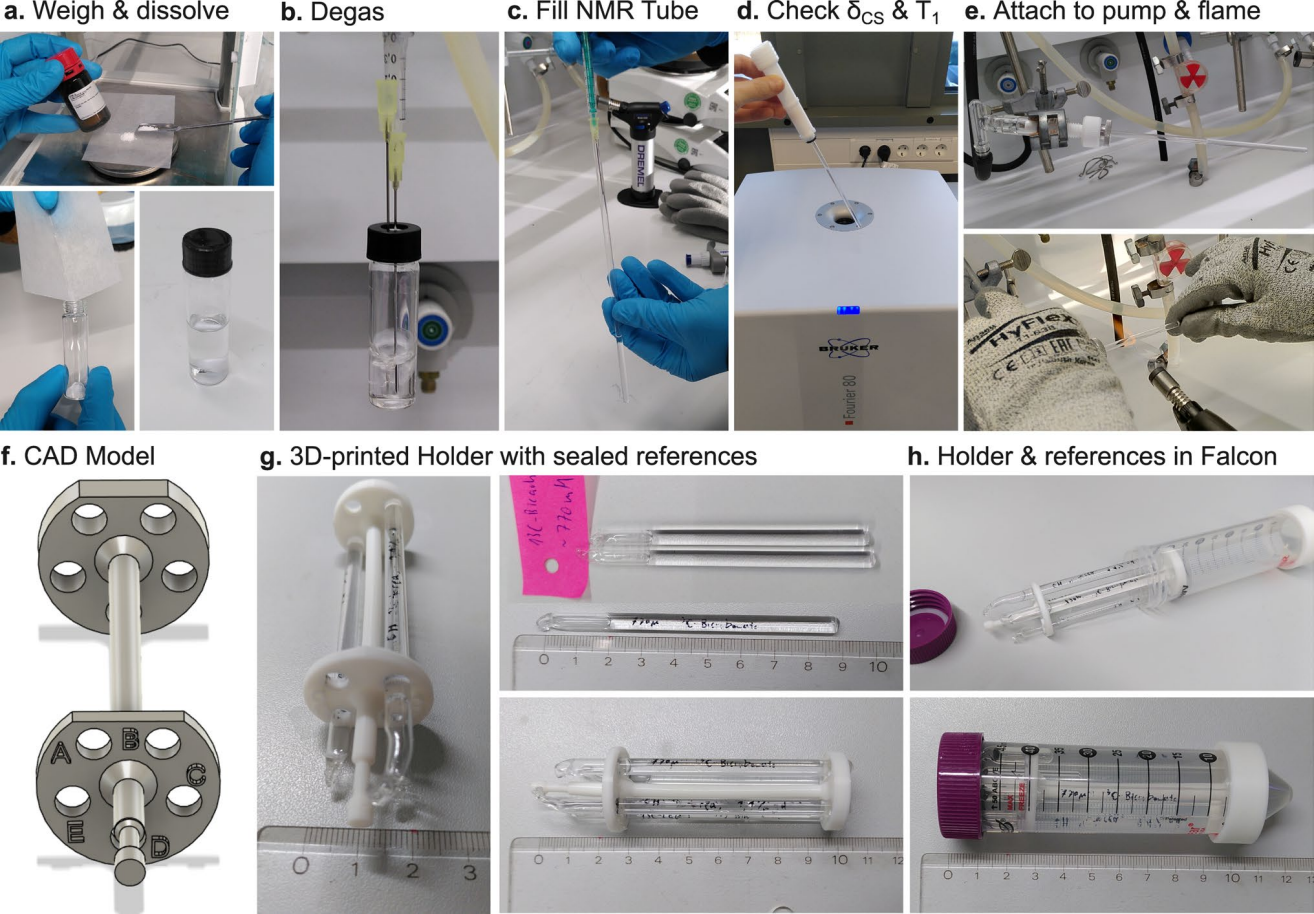
Manufacture of the ^13^C reference solutions by weighing and dissolving the chemicals (**a**), followed by degassing with argon (**b**) and filling into NMR tubes (**c**). After quantification with a tabletop NMR (**d**), T_1_-shortening gadolinium was added, followed by the closing of the vials via vacuumizing and flaming of the tubes (**e**). The final reference solutions in tubes can be inserted into a 3D-printed holder (**f**, **g**), which is placed in a 50 mL centrifuge tube filled with NaCl solution (**h**)

The references for the phantom were produced with the following steps (see also Fig. 1):

- **Step 1:** Alanine, urea and bicarbonate were each dissolved in 2 mL H_2_O, while lactate and ethyl acetate were delivered as liquids and directly used in this form. The ^13^C compounds were each degassed with argon for 5 min at a flow of 100 mL/min and filled into commercially available 5 mm outer diameter NMR vials to create the final volumes/concentrations per vial: 900 μL/1.25 M (bicarbonate), 900 μL/6.06 M (urea), 800 μL/1.75 M (alanine), 1097 μL/10.21 M (ethyl acetate), and 700 μL/4.42 M (lactate). A list of chemicals is given in Table 1.
- **Step 2:** Gadolinium was added to urea and alanine, and the shortening of the longitudinal relaxation time T_1_ was verified using NMR spectroscopy.
- **Step 3:** The NMR tubes were attached to the vacuum pump operating at a slight underpressure of 800 mbar and sealed by carefully flaming the glass at a length of approximately 90 mm. When slowly heated, the melting glass in combination with the underpressure will self-seal, creating an air and liquid-tight closure of the reference vial.
- **Step 4:** The sealed vials and the 3D-printed holder were placed in a regular 50 mL centrifuge tube filled with 0.9% saline (NaCl) solution for measurements at the MRI. Reference tube holders were designed as two thin circular plates of 25/26.5 mm diameter at a longitudinal distance of ~ 65 mm, connected by a 4.5 mm-thick cylindrical structure. Both plates contain holes of 5.1 mm diameter to hold the NMR tubes in position and a 1.7 mm notch to allow air bubbles to pass. The composition of the phantom can be changed between MRI studies according to the experimental requirements.

The relaxation times T_1_ and T_2_ were determined on different systems with varying field strengths for all ^13^C reference solutions. Further experiments were conducted at 11.7 T, including ^13^C spectroscopy, ^13^C/^1^H imaging of the phantom, and spectral separation of the metabolites via chemical shift imaging (CSI). All measurements were performed at the air-conditioned room temperature of the respective facilities (21 °C ± 1 °C), reflecting the expected experimental conditions during routine use of the phantom on imaging systems.

For spectroscopy and imaging experiments at 11.7 T, the following reference solutions were selected to be placed in the phantom: sodium L-lactate-1-^13^C solution, L-alanine-1-^13^C (doped), ethyl acetate-1-^13^C (doped), urea-^13^C (doped), and sodium bicarbonate-^13^C. While lactate, alanine, and bicarbonate are all involved in pyruvate metabolism, urea was chosen since it is commonly used as a reference for frequency adjustments. Note that ethyl acetate-1-^13^C is used as a substitute for pyruvate-1-^13^C, as the latter is unfortunately not suitable for producing a stable reference phantom.

### 1.4 T

Relaxation times at 1.4 T were measured in individual 5 mm NMR tubes using a Spinsolve60 Tabletop NMR (Magritek, Wellington, New Zealand). T_1_ relaxation times were measured using the vendor-provided CarbonT1 pulse sequence. The sequence applies a nuclear Overhauser enhancement (NOE) preparation period, followed by a variable inversion delay, a 90° ^13^C excitation pulse (FA), and acquisition under continuous ^1^H decoupling. For each substance, T_1_ relaxation curves were obtained from a series of single-scan experiments with linearly incremented inversion recovery (IR) delays. The number of delay spacings (no. of spacings), number of points per acquisition (no. of points), and the repetition time (TR) were set individually for each metabolite, as listed in Table 2.1. After apodization, Fourier transformation, and zero-order phase correction, each spectrum obtained from the individual IR experiments was integrated, and the resulting peak integral was plotted as a function of IR delay. T_1_ values were extracted by fitting the recovery curves to a mono-exponential function plus offset.

**Table 2.1:** Acquisition parameters for T_1_ measurements at 1.4 T. Reference solutions marked with (d) indicate doping with gadolinium

Acq. parameter	T_1_ Lac.	T_1_ Ala./Ala.(d)	T_1_ Eth.Ac./Eth.Ac.(d)	T_1_ Ur.(d)	T_1_ Bic.
FA [deg.]	90	90	90	90	90
TR [s]	100	200/100	100	100	90
IR delay [s]	[0.1,…,10]	[1,…,100]/[0.1,…,20]	[0.1,…,40]	[0.1,…,10]	[0.1,…,70]
Spacing	Linear	Linear	Linear	Linear	Linear
No. of spacings	10	40/10	10	10	40
No. of points	8192	16,384/8192	8192	8192	8192
Averages	1	4/1	1	1	4
Scan duration	16 m 53 s	21 h 26 m/17 m 3 s	17 m 23 s	16 m 53 s	4 h 5 m

The T_2_ relaxation times of the ^13^C nuclei at 1.4 T were determined at the Tabletop NMR using the vendor-provided CarbonT2Bulk pulse sequence. The sequence consists of an initial 90° ^13^C excitation pulse, followed by a train of refocusing 180° pulses, during which spin echoes are recorded. For each substance, echo trains were acquired with a fixed echo time (TE), repetition time (TR), number of acquired points per echo (no. of points), and a defined number of echoes (no. of echoes), as summarized in Table 2.2. Echo amplitudes were plotted as a function of echo time, and T_2_ values were obtained by fitting the decay of the echo envelope to a mono-exponential function plus offset.

**Table 2.2:** Acquisition parameters for T_2_ measurements at 1.4 T

Acq. parameter	T_2_ Lac.	T_2_ Ala./Ala.(d)	T_2_ Eth.Ac./Eth.Ac.(d)	T_2_ Ur.(d)	T_2_ Bic.
FA [deg.]	90/180	90/180	90/180	90/180	90/180
TE [ms]	4	10/4	10	4	6
TR [s]	5	5	5	5	5
No. of echoes	2000	4000/2000	4000	500	2000
No. of points	32	32	32	32	32
Averages	1	1	1	1	1
Scan duration	8.23 s	40.36 s/8.24 s	40.27 s/40.26 s	2.20 s	12.25 s

### 3 T

All measurements at 3 T were carried out on a 3 T Philips Ingenia MRI system (Philips, Best, Netherlands) with a four-channel ^13^C mouse thorax phased array coil (RAPID Biomedical GmbH, Rimpar, Germany). Non-localized spectroscopy experiments were conducted independently on the single reference tubes, using only the 3D-printed holder without a surrounding centrifuge tube filled with NaCl to fit the inner diameter of the array coil. T_1_ quantification was carried out using an inversion recovery (IR) sequence without spatial encoding, based on a turbo spin echo (TSE) readout with a 180° inversion pulse, followed by 90° and 180° refocusing pulses (FA). The repetition time (TR) was chosen sufficiently long to ensure full relaxation between acquisitions. Inversion delays were distributed with logarithmic or linear spacing, covering the expected relaxation curve with an appropriate number of spacings (no. of  spacings). After summation over all coil elements, the absolute data values were plotted as a function of IR delays, and T_1_ was estimated by fitting the recovery curves to a mono-exponential function plus offset. A full list of sequence parameters can be found in Table 3.1.

**Table 3.1:** Acquisition parameters for T_1_ measurements at 3 T

Acq. parameter	T_1_ Lac.	T_1_ Ala./Ala.(d)	T_1_ Eth.Ac./Eth.Ac.(d)	T_1_ Ur.(d)	T_1_ Bic.
TE [ms]	5.7	29.1/5.7	2	5.7	5.7
TR [s]	30	100/30	100	30	60
IR delay [s]	[0.1,…,10]	[0.1,…,10]/[0,…,10]	[0,…,50]	[0,…,10]	[0,…,10]
Spacing	Log.	Log.	Log.	Log.	Log.
No. of spacings	16	16	28	16	16
Averages	32	8/32	1	32	32
Scan duration	4 h 16 m	3 h 33 m/4 h 16 m	46 m 40 s	4 h 16 m	8 h 32 m

T_2_ quantification at 3 T was performed using a turbo spin echo (TSE) sequence without spatial encoding. Due to system constraints, the effective maximal echo time (TE_eff_) was limited to a maximum of 2 s. The repetition time (TR) was chosen sufficiently long to ensure complete relaxation between acquisitions. The echo spacing (∆TE) and the total number of echoes (no. of echoes) were selected to capture the exponential decay of the signal amplitude over time. After summation of signals over all coil elements, the echo amplitudes were plotted as a function of echo time, and T_2_ values were obtained by fitting the decay of the echo envelope to a mono-exponential function plus offset. A complete overview of sequence parameters is provided in Table 3.2.

**Table 3.2:** Acquisition parameters for T_2_ measurements at 3 T

Acq. parameter	T_2_ Lac.	T_2_ Ala./Ala.(d)	T_2_ Eth.Ac./Eth.Ac.(d)	T_2_ Ur.(d)	T_2_ Bic.
FA [deg.]	90/180…	90/180…	90/180…	90/180…	90/180…
TR [s]	60	100/60	100	20	60
TE_eff_ [ms]	2000	2000	2000	401	2000
∆TE [ms]	25.8	173.9/25.8	173.9	4.4	37.4
No. of echoes	154	22/154	22	183	106
Averages	512	1/512	1	512	512
Scan duration	8 h 32 m	100 s/8 h 32 m	100 s	2 h 50 m	8 h 32 m

### 11.7 T

11.7 T measurements were carried out on a dedicated small animal MRI system (BioSpec 117/16-USR, Bruker, Germany) with a quadrature, 35 mm i.d., dual-tuned ^13^C/^1^H MR coil (Rapid Biomedical GmbH, Germany). T_1_ mapping was performed with a spin echo-based method, using T_1_-weighted images with varying TR. After an initial 180° inversion recovery pulse, one image is acquired with a Spin Echo sequence with echo time TE fixed for all individual T_1_ experiments. This was done for five separate images using incremented TR values. The range of TR was selected such that it covered the estimated T_1_ range of metabolites (Est. T_1_). The resulting image series represents sampling points along the longitudinal relaxation curve, which was subsequently fitted voxel-wise to extract quantitative T_1_ maps directly in the Bruker TopSpin software. Acquisition parameters (slice thickness, field of view (FOV), matrix size, resolution) were matched to the phantom dimensions, and the full T_1_-Map-RARE parameters are listed in Table 4.1.

**Table 4.1:** Acquisition parameters for T_1_ measurements at 11.7 T

Acq. parameter	T_1_ Lac.	T_1_ Ala./Ala.(d)	T_1_ Eth.Ac./Eth.Ac.(d)	T_1_ Ur.(d)	T_1_ Bic.
FA [deg.]	90	90	90	90	90
TE [ms]	5.53	5.53	5.53	5.53	5.53
TR [s]	[0.8,…,50]	[0.8,…,2500]/[0.8,…,25]	[0.8,…,2500]	[0.8,…,2500]	[0.8,…,50]
No. of T_1_-Exp.	5	5	5	5	5
Est. T_1_ [ms]	10,000	50,000/5000	50,000	50,000	10,000
Rare factor	2	2	2	2	2
Spacing	Log.	Log.	Log.	Log.	Log.
Slice thickness [mm]	25	25	25	25	25
FOV [mm]	30 × 30	30 × 30	30 × 30	30 × 30	30 × 30
Matrix	45 × 45	45 × 45	45 × 45	45 × 45	45 × 45
Resolution [mm]	0.67 × 0.67	0.67 × 0.67	0.67 × 0.67	0.67 × 0.67	0.67 × 0.67
Averages	15	5/15	5	5	15
Scan duration	7 h 2 m	11 h 18 m/3 h 40 m	11 h 18 m	11 h 18 m	7 h 2 m

For T_2_ quantification at 11.7 T, a multi-slice multi-echo (MSME) sequence was employed. This spin echo–based method acquires a series of echo images (no. of echo images), each corresponding to a different echo time (TE_eff_). Following an initial 90° excitation pulse, a train of 180° refocusing pulses generated multiple spin echoes at incremented echo times. Each acquired image thus represented one sampling point on the transverse relaxation decay curve. By fitting the signal intensity of the echo images as a function of TE to a mono-exponential decay plus offset, quantitative T_2_ maps were obtained. The analysis was performed in TopSpin, where regions of interest (ROIs) were defined for each metabolite. A complete list of parameters, including repetition time (TR), slice thickness, FOV, matrix size, and resolution, is presented in Table 4.2.

**Table 4.2:** Acquisition parameters for T_2_ measurements at 11.7 T

Acq. parameter	T_2_ Lac.	T_2_ Ala./Ala.(d)	T_2_ Eth.Ac./Eth.Ac.(d)	T_2_ Ur.(d)	T_2_ Bic.
FA [deg.]	90	90	90	90	90
TE_1_ [ms]	40	100/50	100	6.5176	40
TR [ms]	60,000	150,000/60,000	150,000	60,000	60,000
No. of echo images	20	30/40	30	20	20
TE_eff_ [ms]	[40,…,800]	[100,…,3000]/[50,…,2000]	[100,…,3000]	[6.52,…,130.35]	[40,…,800]
Spacing	Linear	Linear	Linear	Linear	Linear
Slice thickness [mm]	30	30	30	30	30
FOV [mm]	30 × 30	30 × 30	30 × 30	30 × 30	30 × 30
Matrix	45 × 45	45 × 45	45 × 45	45 × 45	45 × 45
Resolution [mm]	0.67 × 0.67	0.67 × 0.67	0.67 × 0.67	0.67 × 0.67	0.67 × 0.67
Averages	13	8/13	8	12	13
Scan duration	9 h 45 m	15 h/9 h 45 m	15 h	9 h	9 h 45 m

For spectroscopic characterization and image-based separation, free induction decay (FID), point-resolved spectroscopy (PRESS) and CSI sequences were employed. The FID sequence consisted of a single excitation pulse (FA) followed by signal acquisition, whereas PRESS applied a 90° excitation pulse combined with two 180° refocusing pulses to achieve spatial localization within a defined voxel (voxel size), fitting the size of the reference vials. For both sequences, the repetition time (TR) was chosen to allow full relaxation, while averaging was applied to improve SNR. The CSI parameters were adapted to yield a reasonable scan duration combined with sufficient spatial and spectral resolution. In all cases, the excitation pulse frequency (pulse center) was centered on ethyl acetate, while the bandwidth for readout and excitation was chosen to cover all resonances. The detailed acquisition parameters are summarized in Table 5.

**Table 5:** Acquisition parameters of FID and PRESS applied for ^13^C spectroscopy at 11.7 T

Acq. Parameter	FID	PRESS	CSI
FA [deg.]	90	90/180/180	10
Spectral samples	2048	2048	512
Spectral width [kHz]	5	5	5
TE [ms]	–	8.13/7.08	1.1
TR [ms]	175,000	175,000	105
Voxel size/resolution [mm]	–	6 × 6 × 32	0.5 × 0.5
Averages	16	16	1
Scan duration	46 m 40 s	46 m 40 s	7 m 10 s
Pulse center [ppm]	171	171	171

### SNR quantification

Signal-to-noise ratio (SNR) values were quantified for the single metabolites of ^13^C FLASH (Fig. 3) and ^13^C CSI (Fig. 4) acquisitions. It was defined as.

$$SNR = 0.66\frac{{\overline{S}}}{{\sigma \left( {noise} \right)}}$$

The SNR was evaluated for all metabolites in magnitude images using MATLAB (version 2024a, MathWorks, Natick, MA). ROIs were segmented for each metabolite and sequence manually, yielding the signal mean $\overline{S}$. The noise standard deviation σ(noise) was computed correspondingly from an ROI lying in the outside corner of the images to ensure artifact-free pure noise contribution.

## Results

**Fig. 2:**
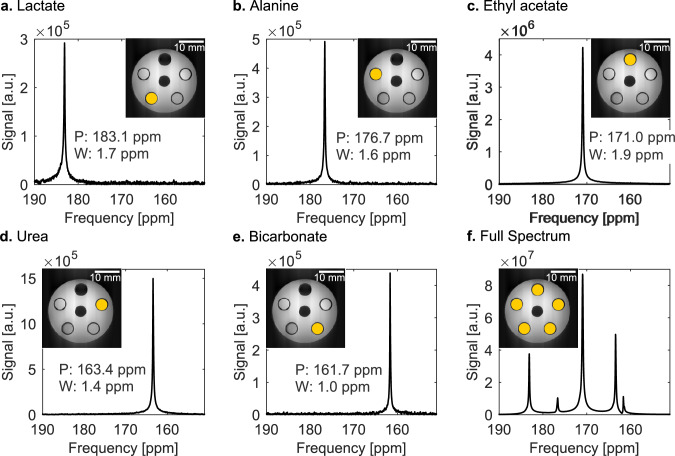
Individual spectra measured at 11.7 T from sodium L-lactate 1-^13^C solution (**a**), L-alanine-1-^13^C (**b**), ethyl acetate-1-^13^C (**c**), urea-^13^C (**d**), and sodium bicarbonate-^13^C (**e**), each labeled with peak position P and full width at half maximum W. The ^1^H MRI panels visualize each reference position inside the phantom. A non-localized spectroscopic measurement of all references combined (**f**) yields all relevant resonance signals together

Individual ^13^C spectra of the phantom reference substances were acquired, confirming distinct and well-separated resonance signals for each metabolite (Fig. 2a–e). Peak positions (P) and linewidths at half maximum (W) were determined, ensuring clear identification of all references. A combined non-localized spectrum (Fig. 2f) displayed all relevant resonances simultaneously.

Table 6 summarizes the composition of the reference vials, including compound volumes, concentrations, chemical shifts, and the measured relaxation times T_1_ and T_2_ at different magnetic field strengths. The relaxation constants were measured longitudinally over twelve months. Longitudinal changes were limited to the known measurement uncertainty. No significant trend over time was observed, indicating a chemical and physical stability as expected for a closed system.

**Table 6:** ^13^C-labeled compounds, their volumes, and the final concentrations that were used to produce the phantom reference vials and to create signals at the respective chemical shifts. The field-dependent ^13^C relaxation constants T_1_ and T_2_ of all references were measured at 11.7 T, 3 T, and 1.5 T

References solution	Vol. [μL]	Conc. [mol/L]	CS [ppm]	11.7 T		3 T		1.4 T	
				T_1_ [s]	T_2_ [s]	T_1_ [s]	T_2_ [s]	T_1_ [s]	T_2_ [s]
Lactate	700	4.42	183.13	2.546 ± 6e − 3	0.89 ± 0.01	2.91 ± 0.15	1.35 ± 0.02	3.38 ± 0.21	1.472 ± 7e − 3
Alanine	800	1.75	176.64	17.45 ± 0.56	2.11 ± 0.02	26.01 ± 0.84	1.68 ± 0.10	35.65 ± 0.83	8.71 ± 0.03
Alanine (doped)	800	1.75	176.64	3.57 ± 0.04	0.81 ± 0.05	3.10 ± 0.11	1.51 ± 0.02	4.35 ± 0.49	1.49 ± 0.01
Ethyl acetate	1097	10.21	170.95	34.70 ± 0.21	3.35 ± 0.02	18.76 ± 1.03	15.54 ± 0.14	36.19 ± 2.27	28.56 ± 0.05
Ethyl acetate (doped)	1097	10.21	170.95	34.49 ± 0.25	3.12 ± 0.02	18.70 ± 1.10	1.33 ± 0.10	29.59 ± 2.06	26.47 ± 0.04
Urea (doped)	900	6.06	163.42	3.14 ± 0.02	0.116 ± 1e − 3	2.96 ± 0.14	0.144 ± 2e − 3	3.96 ± 0.21	0.174 ± 1e − 3
Bicarbonate	900	1.25	161.64	10.94 ± 0.29	1.00 ± 0.1	7.00 ± 0.40	2.93 ± 0.10	12.18 ± 0.28	2.47 ± 0.01

For long-term stability of the phantom, the chemical shifts and mass of the single tubes, containing the reference solutions, were regularly determined over a time span of 20 months (Table 7). For ethyl acetate, observations could only be made over a period of 10 months due to a delayed delivery time. The resonance frequencies of the references showed neither absolute nor relative changes over this time period. The vials’ mass was also measured once a month over the entire time period, showing no deviation within the balance’s linearity of ± 30 mg.

**Table 7:** Longitudinal control measurements of the reference solutions’ chemical shifts (CS) over a maximum time span of 20 months (mm/yy), their estimated error, and the measured weights. All chemical shift values are given in ppm

Reference solution	CS 12/23	CS 05/24	CS 10/24	CS 03/25	CS 08/25	∆CS	Weight [g]
Sodium L-lactate 1-^13^C solution	183.16	183.25	183.15	183.15	183.14	0.05	1.92
L-Alanine-1-^13^C (doped)	176.66	176.72	176.63	176.66	176.67	0.04	1.64
Ethyl acetate-1-^13^C (doped)	–	–	170.97	170.96	170.99	0.02	1.91
Urea-^13^C (doped)	163.39	163.48	163.37	163.43	163.46	0.05	2.05
Sodium bicarbonate-^13^C	161.64	161.78	161.70	161.65	161.69	0.06	2.01

**Fig. 3:**
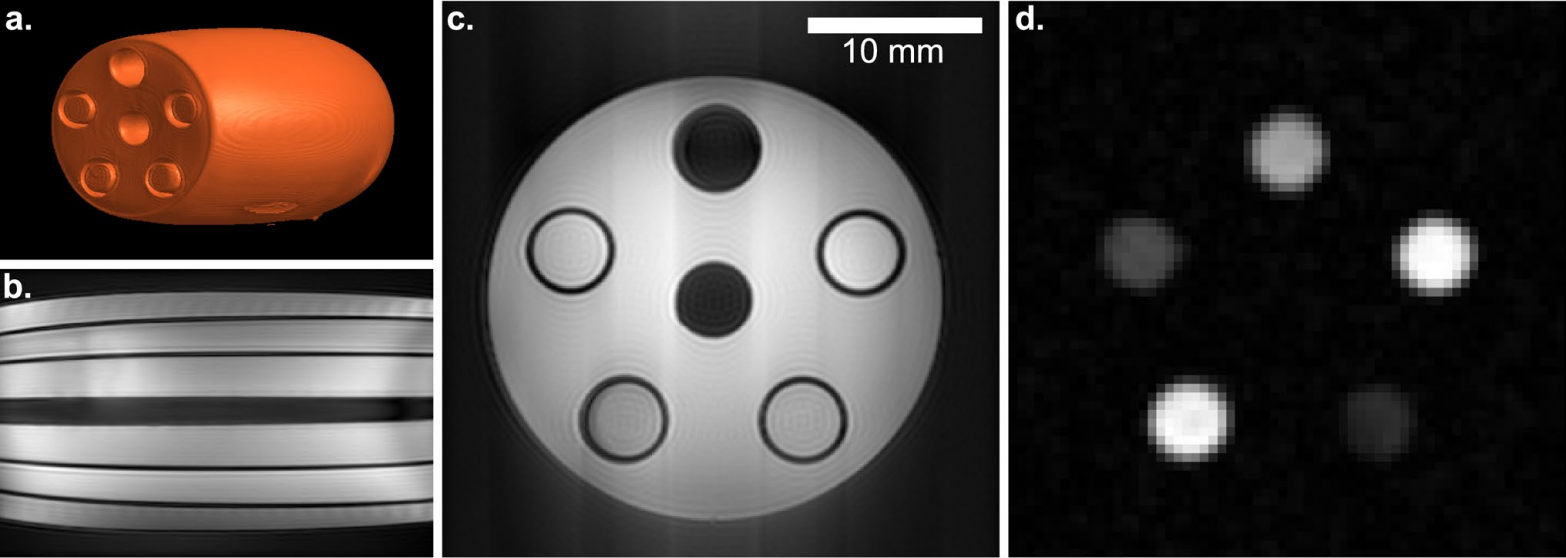
3D rendering (**a**) together with coronal (**b**) and axial (**c**) images of the phantom measured at 11.7 T with ^1^H FISP: FA 8°, TE/TR 2.2/5 ms, resolution 0.25 × 0.25 × 1 mm^3^, FOV 32 × 32 × 64 mm^3^, 8 averages, scan duration 34 m 8 s. An axial 2D slice measured with ^13^C FLASH is shown in (**d**): FA 15°, TE/TR 1.2/100 ms, resolution 0.5 × 0.5 mm^2^, FOV 32 × 32 mm.^2^, slice thickness 32 mm, 64 averages, scan duration 6 m 49 s. SNR calculations of the number-labeled metabolites resulted in values of 60.3 (lactate, 1), 18.6 (alanine, 2), 38.0 (ethyl acetate, 3), 57.9 (urea, 4) and 10.4 (bicarbonate, 5), where numbers indicate the corresponding position in (**d**)

High-resolution structural MR imaging with ^1^H FISP provided clear anatomical visualization of the phantom. The coronal (Fig. 3b), as well as the axial (Fig. 3c) image and the 3D rendering model (Fig. 3a) show homogeneous signals across the phantom in all dimensions. Subsequent ^13^C FLASH acquisitions successfully localized the metabolite signals (Fig. 3d). Quantitative SNR analysis revealed strong signals for lactate (60.3), urea (57.9), and ethyl acetate (38.0), while alanine (18.6) and bicarbonate (10.4) showed comparably lower SNR values.

**Fig. 4:**
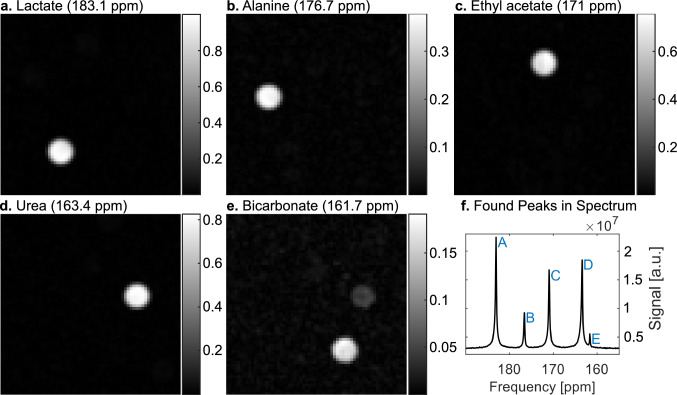
Reconstructed metabolite images of lactate (**a**, SNR 112.4), alanine (**b**, SNR 51.4), ethyl acetate (**c**, SNR 106.2), urea (**d**, SNR 109.6), and bicarbonate (**e**, SNR 23.6) for CSI 2D with a matrix size of 64 × 64, acquired at 11.7 T. Measured with: FA 10°, 512 Spectral Samples, BW 5000 Hz, TR 105 ms, resolution 0.5 × 0.5 mm^2^, FOV 32 × 32 mm^2^, slice thickness 32 mm, scan duration 7 m 10 s, pulse center 171 ppm. **f** shows the accumulated ^13^C magnitude spectrum from all voxels of the acquired data with the corresponding metabolite peaks

Metabolite-specific maps reconstructed from ^13^C CSI measurements demonstrated reliable spectral separation of all five references (Fig. 4a–e), while a small contribution of the urea signal can be identified in the bicarbonate image. The resulting spectra (Fig. 4f) showed distinct peaks for each compound, confirming accurate chemical identification. Among the reconstructed metabolite images, lactate (112.4), urea (109.6), and ethyl acetate (106.2) provided the highest SNR values, while alanine (51.4) and bicarbonate (23.6) yielded lower but still distinguishable signals.

## Discussion

The presented ^13^C MRSI phantom is built from readily available labware, cheap materials that are available in almost all modern laboratories, such as: NMR glass tubes, centrifuge tubes, and 3D-printed structures, which can easily be replaced or renewed. This should allow almost all laboratories to reproduce our recipes and manufacturing instructions.

We used 50 mL centrifuge tubes in our design because of several advantages they offer for preclinical applications. The radial outer diameter of 28–29 mm fits into most small animal volume RF coils. When filled with saline solution around the ^13^C reference NMR tubes, the total volume of ~ 50 mL mimics a coil loading with an animal at a size somewhere between a large mouse and a small rat. Their cylindrical geometry, when aligned along B_0_, is preferable for improved B_0_-field shimming, resulting in low ^13^C signal peak widths and good peak separation. Those tubes come with standardized geometry and dimensions, even when purchased from different manufacturers or suppliers, which simplified the design of the 3D-printed reference tube holders.

The reference tube holders were designed to trap inevitable air bubbles in the saline solution at the front or rear end of the phantom and keep them far away from the MRI FOV. Designing holders that fit various numbers of NMR tubes or can be used with smaller (15 mL) or larger (> 175 mL) centrifuge tubes is simply a matter of CAD design and printing expertise. For multi-center standardization, we recommend using a single standard container and holder design. Therefore, we share our 3D-printable structures for holding NMR tubes as CAD files, which can be downloaded and used under a common open-source license [16]. Beyond preclinical use, the ^13^C reference vials can support potential clinical applications. Placed next to a volunteer in a ^13^C torso coil, they provide a stable chemical standard for calibration, and SNR assessment. This allows simultaneous ^1^H anatomical imaging and ^13^C spectral validation, helping optimize acquisition and reconstruction methods before hyperpolarized ^13^C experiments.

The expensive materials, i.e., the ^13^C labeled metabolite solutions, are to be protected, sealed, and stored under optimal conditions for use as a quality standard for as long as possible, even if the surrounding, cheaper material parts are replaced over time. This raises several challenges. Metabolites tend to have high chemical reactivity; thus, they require a sterile environment and protection from UV radiation. The first issue was resolved by replacing the oxygen with an inert argon atmosphere and adding sodium azide (NaN_3_) before sealing the glass air-tightly via melting. A storage under light-protected and cooled conditions—in our study, in a fridge at 6 °C—did not show any degradation of the ^13^C-labeled materials over months, as would be indicated by changed peak profiles or relaxation properties in the ^13^C spectroscopy. Sub-PPM drifts of the peak positions may arise from temperature or variation in B_0_-field homogeneity. Only pyruvic acid remains too reactive and forms additional molecular structures with itself, yielding a shift of the ^13^C signal peak resonances. With its replacement by 1-^13^C-labeled ethyl acetate, which has a similar chemical shift of 171 ppm, a chemically stable replacement was used in the proposed phantom. In addition, using transparent and UV-light protective glassware, e.g., borosilicate NMR tubes, can potentially extend the stability of the molecular structures, but comes at additional costs. For the sake of completeness, we note that the NMR glass itself can potentially contain impurities at micromolar concentrations, as recently reported [17]. However, using quartz glassware comes at substantially higher cost and would remove impurities that are only noticeable at very strong magnetic fields and high spectral quality—especially ^13^C impurities are presumably low in concentration and signal intensity. No limitations caused by unknown impurities in the ^13^C reference vials were experienced, even at an 11.7 T MRI. Therefore, we suggest using the standard 5 mm outer diameter glassware NMR tubes, which are widely available.

The sealing of the NMR tubes by melting the glass under a Bunsen burner flame should be well practiced in advance, before expensive materials are to be sealed. The inner length of the 50 mL centrifuge tubes requires the NMR glasses to be shortened to 90 mm. The remaining volume of the NMR vials is therefore limited to a maximum of approximately 1.2 mL. The glass melting process, unfortunately, requires a small gas bubble at the top of the liquid as heat insulation; otherwise, the Bunsen flame would boil the ^13^C-labeled solution and potentially destroy the reference material. We found that using a vacuum pump improves the sealing process, but it is not strictly required for airtight sealing. We recommend always testing the freshly closed side of the NMR tube for air and liquid tightness after the glass has cooled off. We found that the molten glass tips of the NMR tubes can be well reinforced against breaking by covering them with two-component epoxy adhesive (e.g., Loctite EA 9492, Henkel, Düsseldorf, Germany). The remaining gas bubbles inside the NMR tubes can cause imperfections in the static B_0_ field. However, if not shaken, the bubble remains in one position within the NMR tube, preferably far outside the MRI FOV, and the gas–liquid susceptibility artifacts can be compensated for with B_0_-field shimming routines.

In addition to sealing, reliable labeling of the NMR tubes is essential to ensure unambiguous sample identification during preparation, storage, and repeated use. We initially used waterproof markers to label the tubes at the glass surface, but the markings wore off over time when phantoms were often inserted into or removed from the holders. A more robust strategy was to number the positions in the 3D-printed holder (e.g., A–E) and track which tube was placed in each slot, which avoids the need to label the glass itself. For long-term storage, placing the tubes in a grid rack with fixed, labeled positions further simplified organization and minimized the risk of confusion. In multi-center or extended studies, additional durable labeling methods such as using nail polish and a color code system, chemical-resistant tapes covered with transparent shrink tubing, or permanent etching/laser marking on the glass, may be beneficial to guarantee that content information and preparation dates remain identifiable throughout extended storage and transport.

Relaxation times at 1.4 T and 3 T were measured in individual 5 mm NMR tubes to provide reference values for multinuclear imaging systems at clinical field strengths. In contrast, the full phantom assembly, including multiple tubes in the centrifuge tube filled with NaCl, was designed for operation at high-field preclinical systems and therefore imaged at 11.7 T. This approach yields relaxation data relevant for multinuclear imaging platforms (1.4 T and 3 T). Additionally, despite the small contribution of the urea signal in the bicarbonate image (Fig. 4e), which is most likely related to the slight resonance peak overlapping in combination with the high concentration difference between the two solutions, it also demonstrates the feasibility of the phantom for spectroscopic imaging experiments at preclinical systems (11.7 T). Further, the results confirm that the reference solutions and overall design are applicable across the range of field strengths encountered in multinuclear MRI and anticipated hyperpolarized ^13^C workflows.

By doping the ^13^C reference solutions with gadolinium, the relaxation time constants T_1_ and T_2_ can be substantially shortened. The reason and advantage of having both, long and short T_1_ and T_2_ for each sample, is for different applications: shortened T_1_ yield increased signals when keeping flip angle, repetition time, and averaging fixed. This is beneficial for MRI ^13^C channel calibration and adjustments for an experiment. Samples with long T_1_ relaxation constants can be used as thermally polarized experimental setups to investigate specialized spectroscopy and imaging methods for later use in HP ^13^C experiments, since in HP ^13^C, a long T_1_ is essential to preserve the non-equilibrium magnetization state over extended time periods. This mimics the realistic signal evolution after dissolution and transfer in hyperpolarization workflows, allowing researchers to evaluate pulse sequences, acquisition strategies, and reconstruction methods under conditions that more closely resemble true in vivo HP experiments. In the present study, relaxation times were measured only at controlled room temperature (21 °C ± 1 °C), reflecting the intended operating conditions for routine phantom use on imaging systems. Although we did not perform a systematic assessment of temperature-dependent relaxation behavior, the expected variation of ± 1 °C in controlled MRI environments is unlikely to cause relevant changes in relaxation times or to limit the usability of the phantom for routine quality assurance.

Compared to previously reported ^13^C phantoms, our approach addresses a complementary but distinct need. Dynamic enzyme-based phantoms [1–3] are valuable for mimicking metabolic conversion processes and cross-site standardization of dynamic acquisitions, but they require complex preparation and often lack long-term stability due to enzymatic degradation. Similarly, phantoms designed for sequence-independent system validation, flip-angle calibration, and coil characterization [5, 6, 12–14] provide vital benchmarks for MRI performance and quality assurance; however, they do not offer a stable chemical reference that can be stored and reused over years. In this sense, our design complements dynamic and metabolite-specific phantoms by providing a durable, reproducible reference standard that can support both preclinical research and multicenter harmonization efforts at low cost.

## Conclusion

We present a simple and reproducible approach for manufacturing a long-term stable ^13^C MR reference phantom using inexpensive, widely available materials such as centrifuge tubes, NMR vials, and 3D-printed holders. The design ensures compatibility with preclinical RF coils, provides appropriate coil loading, and supports robust B_0_ shimming. Open-source CAD files for the holders further enable reproducibility and standardization across laboratories. Stabilization of the ^13^C reference solutions through argon degassing, sodium azide addition, airtight flame sealing, and cooled light-protected storage was validated over up to 20 months, confirming excellent chemical and spectral stability. The substitution of pyruvate with ethyl acetate and optional gadolinium doping expands the range of relaxation properties available for calibration and validation tasks.

Spectroscopic and imaging experiments demonstrated distinct, well-separated resonances with strong SNR values, confirming the phantom’s suitability for MR calibration, method and data validation, and quality assurance. These results represent a successful proof of concept, demonstrating the phantom’s robustness across different experimental conditions and confirming its practical utility. This accessible and sustainable solution provides the ^13^C MRI community with a reliable reference standard for long-term preclinical and clinical studies, supporting reproducibility and comparability across sites and time.

## Data Availability

The datasets used and analyzed in this current study are available from the corresponding author upon reasonable request.
