# Correction: Design and chemical composition of a reference phantom for ^13^C metabolic MRSI

**DOI:** 10.1007/s10334-026-01358-3

**Published:** 2026-05-08

**Authors:** Julian Schüle, Christoph A. Müller, Sebastian Lucas, Leonard Schraff, Stefan Menzel, Tobias Speidel, Ilai Schwartz, Volker Rasche

**Affiliations:** 1 Department of Internal Medicine II, Ulm University Medical Center, 89081 Ulm, Germany; 2 Core Facility Small Animal MRI, Ulm University, 89081 Ulm, Germany; 3 NVision Imaging Technologies GmbH, 89081 Ulm, Germany

**Correction: Magnetic Resonance Materials in Physics, Biology and Medicine** **https://doi.org/10.1007/s10334-026-01330-1**

In the original version of this article, wrong relaxation time values for 3 T were reported in Table 6.

The Table 6 should have appeared as shown below.

**Table 6:** ^13^C-labeled compounds, their volumes, and the final concentrations that were used to produce the phantom reference vials and to create signals at the respective chemical shifts

Reference solution	Vol [μL]	Conc [mol/L]	CS [ppm]	11.7 T		3 T		1.4 T	
				T_1_ [s]	T_2_ [s]	T_1_ [s]	T_2_ [s]	T_1_ [s]	T_2_ [s]
Lactate	700	4.42	183.13	2.546 ± 6e−3	0.89 ± 0.01	3.18 ± 0.01	1.35 ± 0.01	3.38 ± 0.21	1.472 ± 7e−3
Alanine	800	1.75	176.64	17.45 ± 0.56	2.11 ± 0.02	34.20 ± 0.60	6.81 ± 0.03	35.65 ± 0.83	8.71 ± 0.03
Alanine (doped)	800	1.75	176.64	3.57 ± 0.04	0.81 ± 0.05	5.46 ± 0.05	1.58 ± 0.01	4.35 ± 0.49	1.49 ± 0.01
Ethyl acetate	1097	10.21	170.95	34.70 ± 0.21	3.35 ± 0.02	43.80 ± 1.20	27.16 ± 0.14	36.19 ± 2.27	28.56 ± 0.05
Ethyl acetate (doped)	1097	10.21	170.95	34.49 ± 0.25	3.12 ± 0.02	36.80 ± 0.90	21.4 ± 0.08	29.59 ± 2.06	26.47 ± 0.04
Urea (doped)	900	6.06	163.42	3.14 ± 0.02	0.116 ± 1e−3	3.72 ± 0.03	0.125 ± 1e−3	3.96 ± 0.21	0.174 ± 1e−3
Bicarbonate	900	1.25	161.64	10.94 ± 0.29	1.00 ± 0.1	12.99 ± 0.19	2.57 ± 0.02	12.18 ± 0.28	2.47 ± 0.01

The field-dependent ^13^C relaxation constants T_1_ and T_2_ of all references were measured at 11.7 T, 3 T, and 1.5 T. In additional measurements, IR delays for T_1_ scans were increased up to 100 s, while modified TR values were used for T2 quantification.

